# AGE-FRIENDLY ESCAPE ROOM: A POLYPHARMACY QUANDARY

**DOI:** 10.1093/geroni/igae098.3561

**Published:** 2024-12-31

**Authors:** Betsy Yang, Caroline Park, Carter Davis, Joanne Weith, Simeon Kang, Silvia Tee, Christine Gould, Vinita Shastri

**Affiliations:** Palo Alto VA Medical Center, Palo Alto, California, United States; VA Palo Alto Healthcare System, Palo Alto, California, United States; VA Palo Alto Healthcare System, Palo Alto, California, United States; Department of Veterans Affairs Palo Alto, Palo Alto, California, United States; Geriatrics Research and Educational Clinic Center (GRECC), Palo Alto, California, United States; Stanford University School of Medicine, Stanford, California, United States; VA Palo Alto Healthcare System, Palo Alto, California, United States; VA Palo Alto Healthcare System, Palo Alto, California, United States

## Abstract

Gamification, or adding elements like interactivity and puzzle-solving to teaching, has the potential to promote engagement in geriatric medical education. We organized a 60-minute escape room at a regional internal medicine conference to teach the 4Ms (What Matters, Medications, Mobility, and Mentation) Age-Friendly framework to interprofessional providers through gamification. 15 interprofessional participants received a short patient case and formed small teams to “Escape the Room” by rotating through each of the respective stations of 4Ms, moderated by interdisciplinary preceptors, to learn about salient points of geriatric care with the specific “M” in mind for the patient case. At the end of their 4 station rotations, participants received a clue to solve the final puzzle using the 4Ms framework which answers the question “Which medication will we deprescribe first to treat our patient experiencing cognitive impairment, polypharmacy, and falls?” From the retrospective post-then-pre questionnaire, we evaluated participants’ confidence and knowledge of 4Ms and workshop effectiveness. Results showed significant improvement in participants’ confidence (2.73) and knowledge (2.24) of 4Ms (on a 10-point Likert Scale, p< 0.01, n=15, Wilcoxon Signed-Rank Test). Participants strongly agreed with recommending the workshop to others (μ=4.9 out of 5, SD=0.4) and applying 4Ms to their practice (μ=4.9 out of 5, SD=0.3). Qualitative feedback indicated the escape room was both “useful” and “engaging”, though some participants recommended allotting more time to the activity. With this workshop, we demonstrated that an interprofessional escape room can teach important geriatric concepts in an effective and engaging manner.

